# The Role of Extracellular Vesicles in COVID-19 Pathology

**DOI:** 10.3390/cells11162496

**Published:** 2022-08-11

**Authors:** Aline Seiko Carvalho Tahyra, Rodrigo T. Calado, Fausto Almeida

**Affiliations:** 1 Department of Biochemistry and Immunology, Ribeirão Preto Medical School, University of São Paulo, Ribeirão Preto 14049-900, SP, Brazil; 2 Department of Medical Imaging, Hematology, and Oncology, Ribeirao Preto Medical School, University of São Paulo, Ribeirão Preto 14049-900, SP, Brazil

**Keywords:** extracellular vesicles, COVID-19, cytokine storm, vascular dysfunction, immunology

## Abstract

Extracellular vesicles (EVs) have become a trending topic in recent years; they constitute a new intercellular communication paradigm. Extracellular vesicles are 30–4000 nanometers in diameter particles that are limited by a phospholipid bilayer and contain functional biomolecules, such as proteins, lipids, and nucleic acids. They are released by virtually all types of eukaryotic cells; through their cargoes, EVs are capable of triggering signaling in recipient cells. In addition to their functions in the homeostatic state, EVs have gained attention because of their roles in pathological contexts, eventually contributing to disease progression. In the Coronavirus disease 2019 (COVID-19) pandemic, aside from the scientific race for the development of preventive and therapeutic interventions, it is critical to understand the pathological mechanisms involved in SARS-CoV-2 infection. In this sense, EVs are key players in the main processes of COVID-19. Thus, in this review, we highlight the role of EVs in the establishment of the viral infection and in the procoagulant state, cytokine storm, and immunoregulation of innate and adaptive immune responses.

## 1. Introduction

In the last decade, extracellular vesicles (EVs) have been recognized as an intrinsic and crucial mechanism of intercellular communication [1]. Until the last century, EVs were thought to be cell waste, a scenario that has completely changed with recent advances in knowledge about these particles [2]. Extracellular vesicles are secreted by virtually all types of cells from the three domains of life (Bacteria, Archaea, and Eukarya), suggesting that they constitute an evolutionarily conserved mechanism of communication [3]. Extracellular vesicles can be found in biological fluids such as blood, saliva, semen, urine, breast milk, cerebrospinal fluid, peritoneal lavage, and bronchoalveolar lavage fluid (BALF) [4]. Morphologically, they are characterized as nanoparticles consisting of a phospholipid bilayer that carries active biomolecules such as proteins (e.g., enzymes, surface receptors, and ligands), lipids (e.g., cholesterol, phosphatidylcholine, and sphingomyelin), and nucleic acids (e.g., DNA, mRNA, micro-RNA, and long non-coding RNA) [4]. Based on their biological function, biogenesis, size, and content, EVs are classified into three main subtypes: exosomes, microvesicles, and apoptotic bodies [4].

**Figure 1:**
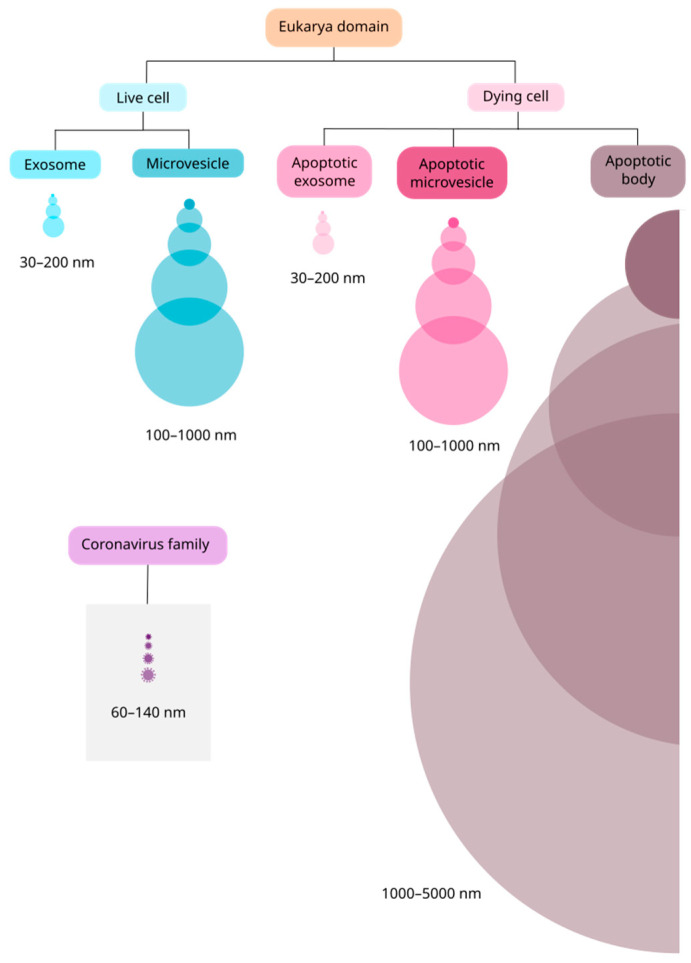
Size comparison between EVs and the coronavirus family. In the Eukarya domain, EVs are secreted by virtually all types of cells, live or dying. Between live cells, there are two main subtypes of vesicles: exosomes and microvesicles. Among cells undergoing apoptosis, there are three main subtypes: apoptotic exosomes (ApoExo), apoptotic microvesicles (ApoMV) and apoptotic bodies. ApoExo and ApoMV were recently described and may have similar size to their correspondents in live cells. The vesicles and the virus are represented proportionally in size.

Exosomes are the smallest vesicles, with a diameter of 30–200 nm (Figure 1) and originate from the endosomal transport route through the inward budding of the membrane of early endosomes. This process is regulated by a protein complex called endosomal sorting complexes required for transport (ESCRT) and its accessory proteins Alix, TSG101, HSC70, and HSP90β, which are commonly used as exosomal markers [4]. The exosomes formed in early endosomes lead to the formation of multivesicular bodies (MVB), which are guided to lysosomal degradation or fusion with the cell membrane in a GTPase-dependent manner [5]. Once fused with the membrane, exosomes are released into the extracellular milieu and exert a wide variety of biological functions by direct ligand-receptor interaction on the surface of recipient cells, by internalization via phagocytosis/pinocytosis, or via fusion with the membrane of recipient cells [5].

In contrast to exosomes, microvesicles result from the outward budding of the cell membrane, and range from 100 to 1000 nm in diameter (Figure 1). Microvesicle biogenesis is still under discussion, but it has been suggested that an influx of calcium and cytoskeleton remodeling, involving actin, microtubules, kinesins, myosins, and SNAREs, participate in this process [4,6]. The main biomarkers used to identify microvesicles are integrins, selectins, and CD40 [5]; however, there is still a need to identify specific markers to distinguish microvesicles from exosomes [4]. As microvesicles are a consequence of the cell membrane budding, their composition, mainly formed by cytosolic and membrane-associated proteins, reflects the physiological state of the cell of origin rather than that of the exosomes [4]. Therefore, understanding the function of microvesicles in homeostatic and pathological states may help understand several disease mechanisms, such as in cancer and viral infections [3,4,5].

Lastly, apoptotic bodies are vesicles released by cells undergoing apoptosis [4], suggesting that this kind of programmed cell death may be “silent” but also comprises an active mechanism of communication sending the “last message” from a cell to its milieu [7]. These vesicles range from 1000 to 5000 nm in diameter (Figure 1) [4,5] and emerge during membrane protrusion because of an increase in hydrostatic pressure followed by actomyosin-mediated cell contraction [8]. Their charge can range from nuclear fragments to entire organelles, such as mitochondria or endoplasmic reticulum [7]. The vesicular membrane permeability integrity is lost, allowing intracellular proteins to act in complex extracellular signaling pathways [9], but there is no consensus about a surface marker, as it depends on the cell of origin [10].

Like cells in the homeostatic state, cells undergoing apoptosis can release apoptotic exosomes (ApoExo) and apoptotic microvesicles (ApoMV) [7]. Apoptotic exosome biogenesis is related to the sphingosine-1-phosphate receptor 1 and 3 (S1P1/3) signaling pathway in the early apoptotic phase, in a caspase-3 dependent-manner, for MVB maturation [7]. The known effects of ApoExo are related to a wide range of pathological events, such as autoimmunity, tumor progression, sterile inflammation, and endothelial disorders [7]. The ApoMVs are apoptotic vesicles up to 1000 nm in diameter (Figure 1) and, despite having a biogenesis mechanism similar to that of apoptotic bodies, they are characterized by superior vesicular membrane integrity [7]. These vesicles are usually identified by using lysosomal-associated membrane protein 1 (LAMP1) and HSP70 as markers [7].

Intriguingly, many of the EV features cited above, i.e., those regarding their size, composition, biogenesis, and biological functions, resemble those from viruses [11]. Once this intersection is pointed out, understanding the EV role in viral infection may contribute to improving healthcare, diagnosis, treatment, and therapy. As viruses are intracellular pathogens and share common mechanisms with EVs, they can hijack EV pathways and use them to complete their cycle and disseminate [11,12]. Non-enveloped viruses such as the hepatitis A virus use this strategy to acquire an “envelope” and evade the immune response [11]. Enveloped viruses such as hepatitis C virus may also use the EV pathways to their advantage [13].

In addition to delivering viral particles, EVs released from virally infected cells can carry viral genome and proteins, leading to a dualistic response. That is, whereas these EVs may act as pathogen-associated molecular patterns (PAMPs) and trigger an antiviral response, they also can contribute to facilitating viral transmission [11]. It is still unclear which mechanism leads one response to prevail over the other, and sometimes both responses are detected under infection from the same virus [11,12,14]. Studies involving the association of the human immunodeficiency virus (HIV) and EVs have shown that vesicles can favor viral propagation [15], turn cells more permissive to infection by modulating the expression of receptors [16,17], promote viral stability and replication via host molecules [16,18], and activate latent viruses by uninfected cell EVs [19]. In contrast, vesicles can also trigger the classical antiviral immune response by stimulating the release of proinflammatory mediators in other cells [20,21], in addition to controlling viral replication [22] and impairing propagation [23].

The relevant findings related to this retrovirus, which have also been demonstrated in other relevant viral infections such as that of herpes simplex virus 1 (HSV-1) [24,25,26,27], highlight the relevance of investigating the association of EVs with infections by the coronavirus family members. Such a relationship is not new and has already been described by some authors in the last two decades [28,29]. The coronavirus family comprises enveloped positive single-stranded RNA viruses with a diameter of 60–140 nm (Figure 1) [30,31]. Until 2019, there were six species of coronavirus responsible for human respiratory diseases described in the literature [32]. The seventh species, eventually named SARS-CoV-2, broke out in China in December 2019, leading infected people to a severe acute respiratory syndrome that has caused more than five million deaths in the world in the last two years [31].

Given this COVID-19 pandemic scenery, a scientific race was launched to develop a vaccine [33], leading to a revolution in the vaccinology field. Nonetheless, deepening into the core of the basic scientific research of any disease is a crucial step towards achieving clinical applications such as improved health care or more effective vaccines. Taking this into account, the interplay between SARS-CoV-2 and EVs blooms as a new and promising subject matter whose state of the art will be dissected in this review.

## 2. The Contribution of Extracellular Vesicles for SARS-CoV-2 Infection of Host Cells

The SARS-CoV-2 disease is transmitted via airborne particles and droplets. Once a person is exposed to the virus, it enters the airways via the upper respiratory tract during inhaling [30]. The process of infection of cells involves the viral spike protein and the host receptor angiotensin-converting enzyme 2 (ACE2) [31,34]. Spike is a trimeric transmembrane protein arranged across the envelope surface of the virus; when viewed in electron micrograph images, such arrangement resembles a crown, justifying the etymology of the word “coronavirus” [31]. Because of its molecular affinity for ACE2, the virus may enter pulmonary cells with the help of host serine proteases, such as transmembrane serine protease 2 (TMPRSS2), which cleaves spike, allowing the fusion of the viral particle and the internalization of the viral genome [34]. As ACE2 and TMPRSS2 are highly expressed in alveolar epithelial cells, such as type II pneumocytes and endothelial cells [35], the virus settles in the lungs and promotes an acute respiratory syndrome.

A wide variety of cells in the lungs release EVs, including epithelial cells, endothelial cells, alveolar macrophages, and neutrophils [36,37]. Alveolar macrophages are suggested to be the major source of EVs in BALF during infectious stimuli [38] such as that by SARS-CoV-2. Recently, a study reported the presence of ACE2 in EVs released by some types of cells [39], thereby supporting the participation of these vesicles in facilitating infection, given that ACE2 is a key mediator in the fusion between SARS-CoV-2 viral particles and the host cell membrane. Another important molecule is CD9, a tetraspanin that is abundantly expressed in exosomes; it plays a pivotal role not only in exosome biogenesis but also contributes to viral infection. Once transferred to healthy cells, CD9 turns them susceptible to virus docking [31,36]. Alternatively, coronaviruses may enter cells via the caveolin-1-dependent endocytic pathway [40]. During this process, dynamin is recruited to cut off the neck of the caveolae, allowing internalization of the virus-containing vesicle, which is transported in the cell via actin cytoskeleton remodeling [40]. As caveolin-1 is also a critical regulator of the biogenesis and load selection of EVs [41], it is also part of such loads. It has been suggested that SARS-CoV-2 spread may be facilitated by caveolin-1-expressing EVs [31,36,42].

Intriguingly, after the invasion, coronaviruses have a peculiar replication mechanism through which they induce remodeling of the endoplasmic reticulum of infected cells to form replicative organelle-like structures [43,44,45]. With a complex architecture, these organelle-like structures are formed by a double phospholipid bilayer harboring viral proteins such as nonstructural protein 3 (Nsp3) [43,44] and constitute an active site for viral RNA synthesis [43,44,45]. Recently, a pore was described to be part of this replicative structure; it may constitute an RNA exchange pathway between the inner region and the cytosol, where the RNA will be encapsidated [44]. As this membranous replicative structure contains viral components, it may contribute to the formation and release of EVs at some stage, as reported above for other viruses, although there is still no evidence of the involvement of these structures in EVs biogenesis during SARS-CoV-2 infection.

Although the exact molecular events are still a puzzle, recent evidence suggest that, under symptomatic respiratory infections by coronaviruses and other viruses, there is a significant increase in circulating exosomes expressing lung self-antigens, viral antigens, and 20 S proteasome [46,47]. In addition to allowing viral spread, EVs also may activate host immune responses once they carry viral and self-antigens [31]. The systemic increase in exosomes reported and detected during SARS-CoV-2 infection [48,49] may be closely related to the pathological events characteristic of COVID-19.

Intriguingly, these exosomes are gaining focus once they not only play a pathogenic role in the disease. It was recently demonstrated that exosomes from COVID-19 patients carry ACE2 receptors on its surface and that these vesicles can neutralize SARS-CoV-2 infection in humanized ACE2 (hACE2) transgenic mice by competing with the binding site of cellular ACE2 [50]. The potency of the blockage was calculated to be 135-fold higher than vesicle-free recombinant human ACE2 and the efficacy reached 60- to 80-fold higher in preventing SARS-CoV-2 infection in vivo [50]. These remarkable data show the relevance of the use of these ACE2-exosomes as a therapeutic intervention to block and reduce the infection.

## 3. Pathological Mechanisms of COVID-19 and the Role of Extracellular Vesicles

Although the upper respiratory tract is the primary site of infection for SARS-CoV-2, the disease can lead to severe extrapulmonary clinical manifestations [51]. The spectrum of symptoms ranges from asymptomatic to critical cases and, in more severe cases, death. Among the multisystemic manifestations of COVID-19, vascular dysfunction appears to be a key factor in the pathophysiology of severe illness [51,52]. Such is the relevance of this disturbance in the course of the disease that some authors have suggested that COVID-19 is a “vascular disease” [52]. Indeed, most patients with severe COVID-19 are in a hypercoagulative state that increases the risk of thromboembolic events [49,50,51,52].

To comprehend the mechanisms that underlie the vascular dysfunction, the first aspect is that endothelial cells abundantly express the ACE2 receptor, which leads to virus tropism to blood vessels [52]. Among its physiological functions, the vascular endothelium is the first responder of the host defense [53] and, once homeostasis is disrupted by SARS-CoV-2 infection, endothelial cells undergo a transition process from a resting to an activated state [52]. This process is mainly led by circulating inflammatory molecules, such as interleukin-1 (IL-1), IL-6, tumor necrosis factor-α (TNF-α), PAMPs, and damage-associated molecular patterns (DAMPs), which heighten inflammation in the local environment [51,52]. Consequently, the recruitment of innate immune mediators such as neutrophils contributes to thrombosis by the formation of neutrophil extracellular traps (NETs) [52].

In addition to the positive regulation of proinflammatory gene expression, activated endothelial cells increase the expression of plasminogen activator inhibitor-1 (PAI-1) and tissue factor (TF, also known as CD142), which are two components that contribute to thrombus formation [52,54]. Intriguingly, a clinical study with a cohort of COVID-19 patients revealed that circulating extracellular vesicles express CD142 active molecules, which are closely related to the enhanced procoagulant activity observed in the disease course [54]. This evidence corroborates the findings of Holnthoner et al., who demonstrated, in 2017, the relationship between endothelial cell-derived EVs bearing CD142 and their effects in a prothrombotic state [54]. The TNF-α is a key element in the molecular mechanism involved in this vascular dysfunction [51,52]; TNF-α is usually detected at high levels in the peripheral blood of patients with COVID-19 when compared to healthy donors [48]. Apart from its proinflammatory activity, TNF-α is responsible for triggering the release of EVs by endothelial cells and is correlated with the expression of CD142 in vesicles [54].

Not only endothelial cells but also platelets release EVs that influence the functionality of the vascular system [55]. During COVID-19, increased platelet reactivity and platelet-leukocyte interactions have been reported to contribute to aggregate formation [56,57]. In addition, a significant outgrowth in circulating platelet-derived EVs has been reported [58]; they constitute the major source of CD142 in plasma under homeostatic conditions [59]. This abundance reinforces their role in pathogenesis. Given the direct relationship between circulating platelet-derived EVs and disease severity, these vesicles have been proposed as useful biomarkers for predicting patient outcomes [48,55,58].

The mechanisms involved in this chain of pathological events in COVID-19 are the result of an exacerbation in the homeostatic physiological functions [52]. Along with vascular dysfunction, another pillar of COVID-19 is the overactivation of the immune response [52,60]. During the infection of endothelial and epithelial cells by SARS-CoV-2, there is an overproduction and release of IL-1, IL-6, and TNF-α proinflammatory cytokines that, together with chemokines, activate and recruit innate immune cells [36,61,62]. Neutrophils and macrophages are the protagonists in this first step and, in response to local proinflammatory cytokines, these cells differentiate and acquire an inflammatory profile [36]. This positive feedback amplifies the immune response, culminating in a hyperinflammatory state that reverberates in a cytokine storm [36,60,61,62].

The systemic inflammatory cascade is triggered by a cytokine storm and ends up in a multi-organ dysfunction syndrome to which EVs are tightly related, although their functions have not yet been dissected [36]. Bastarache et al. reported that patients with acute respiratory distress syndrome (ARDS) have higher concentrations of EVs in lung edema fluid than controls [63]. They also noticed that these vesicles are released under inflammatory stimuli, and once incorporated by alveolar macrophages, they induce the production of proinflammatory cytokines, such as IL-6 and TNF-α, contributing to hypercytokinemia [63]. This promotes a robust inflammatory influx in the lungs leading to alveolar damage, suggesting a pathological role for lung EVs in the cytokine storm [34,36,61].

In addition, alveolar macrophage derived-EVs may contribute to the cytokine storm in COVID-19 [36,64]. As previously demonstrated, alveolar macrophages immediately synthesize and release EVs in BALF following LPS instillation in mice [64]. When phenotyping the EV content, TNF was detected in high amounts, whereas IL-6 and IL-1β were found at lower levels, suggesting a proinflammatory EV profile [64]. In summary, these data indicate that, in COVID-19, EVs contribute to the accentuation of vascular dysfunction and cytokine storm, events that converge to the renin-angiotensin-aldosterone (RAAA) axis, exacerbating a set of physiological functions that under tightly regulated conditions would otherwise control the infection [51,52,65].

These physiological and intrinsic functions of the extracellular vesicles can be exploited as therapeutic intervention strategies to improve the recovery of the damage caused by the vascular dysfunction and by the cytokine storm [66]. As demonstrated previously, neutrophils are the first responders at the site of infection. Its derived EVs in early response can exert functions that enhance tissue repair like stimulating the secretion of immunomodulatory cytokines like Tumor Growth Factor-β (TGF-β) by macrophages and downmodulating the secretion of proinflammatory molecules like IL-8, IL-10, and TNF-α [66,67]. Neutrophil EVs also express an important anti-inflammatory glucocorticoid, the annexin 1, which is suggested to dampen cell recruitment by anti-migratory properties of leukocytes [68]. Moreover, endothelial cells-derived EVs may perform protective roles by providing a catalytic cell surface for the conversion of plasminogen into plasmin, leading to fibrinolysis by clot dissolution [66,69]. Altogether, these data highlight the potential use of EVs as a therapeutic intervention in COVID-19 to manage the damage caused by vascular dysfunction and cytokine storm.

## 4. Immunoregulation of Innate and Adaptive Response Mediated by Extracellular Vesicles during COVID-19

In COVID-19, the immune system undergoes a series of changes [36,51]. The most common clinical findings are T-cell lymphopenia and hyperinflammation, which are characterized by high levels of C-reactive protein, D-dimer, ferritin, and other relevant biomarkers [36,51]. In addition to the roles mentioned above, EVs contribute to dysregulation of the immune system through effector activities triggered in innate and adaptive immune cells such as macrophages, monocytes, neutrophils, dendritic cells, B- and T-lymphocytes [51].

An intriguing study has recently shown that exosomes from patients with severe COVID-19 carry proteins related to metabolism, inflammation, and stress response, which correlates with the known pathophysiology of severe cases and might be related to a worse prognosis [70]. This might be explained by the poor immunogenicity of severe patient exosomes compared to those of mild patients due to the low levels of expression of the Spike fragment and the inefficacy to act as an antigen presenting source and to activate CD4+ T-cells [70]. In contrast, patients with mild disease present exosomes carrying proteins related to activation and effector immune mechanisms, which would agree with a better outcome [70,71]. The EVs are found in greater abundance in mild cases as compared to severe patients, and present an enrichment in viral Spike protein fragments, showing higher affinity in binding anti-RBD antibodies [70]. It has been shown that these EVs are a driving force to activate CD4+ T-lymphocytes via MHC class II molecules, eliciting an immune response that cohesively intercommunicate pulmonary epithelial and endothelial cells to reach the recovery status [70,71].

In a proteomic study evaluating temporal changes in EVs in COVID-19, it was demonstrated that the main dysregulated pathways corresponded to the complement system and coagulation cascade with high levels of expression of C1r, C1s, C8γ, fibrinogen-γ, fibrinogen-α, and coagulation factor V, components of innate immune response that, once dysregulated, contributes to the vascular dysfunction and thrombosis [71,72]. These data are in line with the exosome profile of the severe patients cited above [70,71]. The patient, when entering the resolution phase of the disease after facing a hyperinflammatory response, undergoes lipid metabolic changes in vesicles, leading to an increased membrane anisotropy, which reduces exosomal localization of prenisilin-1, allowing the shut-off of the NOTCH-1 signaling pathway and softening of systemic inflammatory response [72]. As the patient progresses to the resolution phase, it is observed the interruption of a positive feedback generated by inflammatory cytokines such as IL-6, a restoration of T-lymphocyte count and an offset of lipidic and proteic dysregulation towards homeostasis [72].

Thus, in this lipidome study led by Lam et al., it was suggested that the presence of oxidized derivatives of lipids in extracellular vesicles may trigger an immunomodulatory and antiviral response [72]. Therefore, EVs can trigger not only pro-inflammatory responses contributing to pathological effects, but also play a role in anti-inflammatory mechanisms performing a protective outcome by promoting tissue repair and remodeling activities [60,73]. This fine-tuned regulation is directly related to the EV profile and the time point during COVID-19 [72]. Its immunomodulatory effects on COVID-19 have been demonstrated in different tissues like kidney, heart, brain, liver, lung and in vessels, evidencing its potential for clinical translation and use as therapeutic treatment [66].

Specially, mesenchymal stromal cell (MSC)-derived EVs are emerging as a potential therapeutic alternative, in addition to the wide use of these cells in clinical trials supported by guidelines of the International Society for Cellular Therapy [74]. Once MSC demonstrates anti-inflammatory, immunomodulatory, anti-fibrotic and antioxidant properties [74,75], the use of its derived EVs may implicate in the improvement of COVID-19 patients’ outcome as they enhance VEGF (Vascular Endothelial Growth Factor), HGF (Hepatocyte Growth Factor), FGF7 (Fibroblast Growth Factor 7), TGF-β signaling pathways, leading to the repair of damaged tissue.

In a very small single-center pilot study with 10 patients with COVID-19 pneumonia, seven patients were intravenously infused with MSC and three received placebos. Those infused with MSC appeared to be correlated with lung function improvement and appeared to ameliorate the clinical status, in consonance with the detection of MSC high expression of the above-mentioned trophic factors [76]. After treatment with MSC, serum IL-10 increased whereas TNF-α significantly decreased, suggesting a reprogramming in the immune response profile, favoring an immunomodulatory phenotype that reverberates in the recovery of even severe patients [76]. The transcriptional analysis reported that MSC-derived exosomes carry microRNAs responsible for impairing the apoptosis cascade by inhibiting PTEN (Phosphatase and Tensin homolog) and PDCD4 (Programmed Cell Death 4) and for suppressing NF-κB signaling pathway leading to a phenotype swift in alveolar macrophages from a pro-inflammatory to an immunomodulatory profile [77].

Despite the encouraging results, evidence prior to the pandemic alerted to potential adverse events related to coagulation. Silachev and colleagues have demonstrated that MSC-derived EVs express annexin V, which implies in the exposition of phosphatidylserine (PS) on their surface, a phospholipid component that acts as an important cofactor of the coagulation cascade [78,79]. In summary, it is crucial to evaluate the biological safety and side effects of the application of vesicles as a therapy in each of the diseases once they have a broad spectrum of action.

## 5. Conclusions

**Figure 2:**
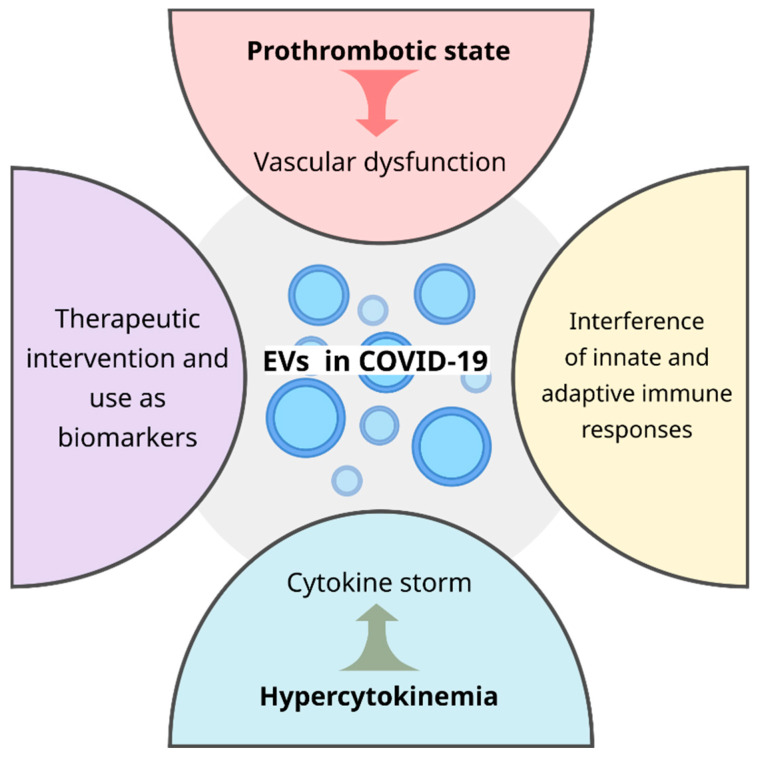
During COVID-19 disease, EVs are key players in the vascular dysfunction, cytokine storm, innate and adaptive immune responses and may have a potential application as therapeutic intervention and as a non-invasive biomarker, improving the clinical outcome of patients.

In summary, EVs are a new branch of knowledge in vogue in the context of several diseases given the wide variety of roles played. In COVID-19, EVs contribute to a prothrombotic state leading to vascular dysfunction, heightening hypercytokinemia which ends up in a cytokine storm, and interferes in the innate and adaptive immune response (Figure 2). In addition to deepening studies on its influence on pathogenic conditions, its potential as a therapeutic intervention strategy and use as a biomarker still needs to be explored. Certainly, what is known about extracellular vesicles in COVID-19 today is the tip of the iceberg of the immense knowledge that is still required to clarify this promising field and thus bring to light well-designed EV-based clinical interventions.
